# Supplementary material for: Chromothripsis during telomere crisis is independent of NHEJ, and consistent with a replicative origin
Source: Genome Res. 2019 May;29(5):737–49. doi: 10.1101/gr.240705.118 (PMC6499312; doi:10.1101/gr.240705.118)
Supplement: Supplemental Material [file supp_gr.240705.118_Supplemental_file_1.zip › contigs/annotated_contigs/DB47/contig.2.DB47_length_293_mean_cov_5.08532423208.docx]

**DB47_length_293_mean_cov_5.08532423208**

```
TTTTCTATTTTTAGTAGAGACAGGCTTTCACCAATTTGGCCAGGCTGGTCTTGAACTCCTAACCTCAGGTGATCTACCTGCCTT|GGCC
 >chr17:40710563-40710670 - E=5e-35 p=0e+00                                          >ch
TCCCAAAGTGCTGGGATTA|CAGGCGTGAGCCACCAGGCCCGGCCCGTTTTTTAATTTTTCTTTATAATACTAAAATTAAATTAAATTA
r19:4885272-4885481 - E=1e-105
AATTTTAAAAAGACTGGTCCAGGGTTTGTCTTCTTGGGGGAATGGCCCTGAAATACTTCTCCAGCCACCTTCGAACTGCAAGATACTGG

TACCTCCCTCCCTCTTGCTGCAGGGTGT
```
